# Polymorphisms of chemokine and chemokine receptor genes in idiopathic immune-mediated posterior segment uveitis

**Published:** 2007-03-23

**Authors:** Muhammad A. Ahad, Tom Missotten, Atiyeh Abdallah, Penny A. Lympany, Susan Lightman

**Affiliations:** 1 Institute of Ophthalmology and Moorfields Eye Hospital, Imperial College, London, UK; 2 Moorfields Eye Hospital, Imperial College, London, UK; 3 National Heart and Lung Institute, Imperial College, London, UK

## Abstract

**Purpose:**

Chemokines are important inflammatory mediators that play a crucial role in uveitis. Polymorphisms in chemokine genes can alter the expression of these genes in the inflammatory cells, which, in turn, can affect the clinical phenotype of the disease. The purpose of this study was to identify polymorphisms in chemokine genes that can predict visual outcome in patients with immune-mediated posterior segment uveitis.

**Methods:**

This is a case-control study of 141 Caucasians with idiopathic immune-mediated posterior segment uveitis and 282 controls matched by age and ethnicity. Six polymorphisms in four genes, (MCP-1-2518A/G, RANTES-403G/A, RANTES-28C/G, CCR2 V64I, CCR5-59029G/A, and CCR5 32 bp deletion) were analyzed by sequence specific primers polymerase chain reaction.

**Results:**

Patients with G allele at MCP-1-2581 developed the disease at an early age as compared to patients with A allele corrected p value pc=0.003. Also patients with A allele at RANTES-403 position developed less severe disease and had better visual outcome when compared with patients with G allele (pc=0.02) Final visual acuity after 18 months was better in patients with 32 bp deletion of the CCR5 gene and in patients with the CCR2 wild-type genotype pc=0.02 and pc=0.04, respectively. Patients with the CCR2 64I allele also had a higher risk of developing an elevated intraocular pressure as compared to patients with the wild-type genotype (pc=0.007).

**Conclusions:**

Though the utility for prediction of disease susceptibility of the studied polymorphisms in chemokine genes is in general not robust, we have found that polymorphisms in chemokine genes can influence the outcome of patients with idiopathic immune-mediated posterior segment uveitis. These associations require further analysis in other groups of patients.

## Introduction

Uveitis is traditionally described as an inflammation of the uveal tract and anatomically categorized as anterior, intermediate, posterior, or pan uveitis. However, other structures including the retina and vitreous are frequently involved in the inflammatory process. It is a relatively common inflammatory eye condition with reported annual incidence between 11.4 and 52.4 per 100,000 and prevalence between 38 and 730 per 100,000 [1-5]. Uveitis predominantly affects patients in the working age group, that is between 21 to 60 years [6]. Despite current advances in diagnosis and management, visual loss occurs in 35-40% of patients with uveitis [7].

Although anterior uveitis is the most common form of intraocular inflammation, intermediate, posterior and pan uveitis have an increased incidence of visual morbidity. These are characterized by inflammation of choroid, retina, retinal vessels, vitreous, and ciliary body either in isolation or as a part of pan uveitis. For this group the term posterior segment intraocular inflammation (PSII) has been adopted [8]. PSII or posterior segment uveitis (PSU) can be either infectious or immune-mediated in origin. Immune-mediated PSII accounts for 22% to 38% of all cases of uveitis seen in tertiary care centers [9-12]. The term covers a range of clinical phenotypes, such as idiopathic intermediate uveitis and birdshot retinopathy, but all share the same underlying immune etiology and are immuno-phenotypically similar [13]. An animal model for PSU has been developed that has greatly increased our knowledge of immunopathology and various subgroups involved in this group [14]. Immune-mediated PSII is broadly divided into two categories depending upon the presence of associated systemic disease. Patients with sarcoidosis, Behcet's disease, or multiple sclerosis can develop PSII, and each manifestation has its own clinical and immunopathological features. However, patients with idiopathic intermediate uveitis, white dot syndromes, and multifocal choroiditis share the same pathology, have no associated systemic features, and are considered to have idiopathic posterior segment uveitis (IPSU). Studies have shown the inflammatory process in IPSU is perpetuated by a Th1-mediated inflammatory response [13-16], but what triggers the uveitis initially and why certain groups of people are more susceptible than others is not known.

The genetic predisposition to develop uveitis has long been investigated. The association of HLA genes, particularly HLA-A29 in birdshot chorioretinopathy and HLA-B27 in anterior uveitis, is well known, but the mechanisms of disease induction remain elusive. Recently, attention has been focused on single nucleotide polymorphisms (SNPs) in the genes controlling the inflammatory processes in immune-mediated diseases. Some SNPs may alter protein structure and function through a single nucleotide base substitution in a gene's coding region, or may increase or decrease gene expression either by affecting mRNA stability when occurring in a gene's 3' untranslated region or by altering transcription factor binding when occurring in the 5' promoter region.

Chemokines, which are chemo-attractive cytokines, are one type of the many proteins involved in the inflammatory process. They have been shown to play a critical role in the development of inflammation in diverse immune-mediated disease models [17-19]. Similarly their role in ocular inflammation is becoming more clear; they have been shown to be produced by ocular cells cultured in vitro, and monocyte chemoattractant protein-1 (MCP-1, now known as CCL2), RANTES (CCL5) and interleukin-8 (IL-8) have been detected in ocular fluids and tissues during intraocular inflammation [20-25]. Human retinal pigment epithelial cells (RPE) and retinal microvascular endothelial cells are also able to produce these chemokines [23,26-28].

Animal studies of PSU have shown that CCR5 is a receptor of various chemokines like RANTES, MIP-1α, and MIP-1β plays a crucial role in the migration of Th1 cells to the site of inflammation [29]. Increased expression of CCR2 (receptors for MCP-1/CCL2) and CCR5 genes on posterior segment extracts have also been found in animal models of posterior uveitis [30].

Quite a few functional polymorphisms have been discovered in these chemokine genes particularly in the promoter regions. Promoter polymorphisms at the -2518A/G position of the MCP-1 gene [31] -28C/G and -403G/A of the RANTES gene [32,33], and -59029G/A of the CCR5 gene [34] have been shown to affect the expression of genes and have been noted to affect the phenotype of various immune-mediated diseases. A SNP at position 46295 has been identified in the open reading frame of CCR2. The G/A substitution leads to replacement of valine by isoleucine at amino acid 64 (CCR2 V64I) in the first transmembrane domain of CCR2 [35]. Although this change is considered to be non-functional, it has been found to be associated with various immune-mediated diseases particularly sarcoidosis [36]. Another widely reported polymorphism is the CCR5 32 bp deletion mutation (del. 32) within the coding sequence of the extracellular loop of the CCR5 gene. This polymorphism also affects gene expression [37] and has been noted to be protective in many immune-mediated diseases [38,39].

Although many studies have demonstrated association of these SNPs with various immune-mediated diseases, such as sarcoidosis and Behcet's, the evidence in uveitis is lacking. At least two studies have investigated the role of MCP-1-2518A/G, RANTES-28C/G, -403G/A and CCR5 del. 32 in uveitis in Behcet's disease [40,41], but no particular association has been identified. To explore the role of chemokine SNPs in IPSU, we examined whether these six polymorphisms (MCP-1-2518A/G, RANTES-28C/G and -403G/A, CCR2 V64I, CCR5-59029G/A, and CCR5 del. 32) were associated with IPSU. The aim was determine whether these polymorphisms can affect disease phenotype and predict visual outcome and response to treatment.

## Methods

This study was approved by the Ethics Committee of Moorfields Eye Hospital and was conducted according to the tenets of the Declaration of Helsinki. After giving informed consent, patients with IPSU were recruited from the uveitis clinic at Moorfields Eye Hospital in London. IPSU was defined as a noninfectious inflammation of the choroid, retina, retinal vessels, vitreous, or ciliary body in the absence of any known systemic disease. By definition, this excluded patients with sarcoid associated uveitis, Behcet's-associated uveitis, Vogt-Koyanagi-Harada disease as well as patients with multiple sclerosis or any other identified systemic disease. A complete ophthalmic examination was undertaken that included valuation of visual acuity, intraocular pressures, dilated fundus, and an anterior segment slit-lamp examination. Where necessary, fundus fluorescein angiography, optical coherence tomography, B scan, and visual field tests were performed.

Clinical details were obtained for each patient and included the following: age of onset, follow up period, laterality of disease, type and pattern of posterior uveitis, visual acuity, and ocular complications that can reduce vision such as cataract, glaucoma, cystoid macular edema (CME), optic atrophy, and retinal detachment. We also took into consideration the duration and amount of systemic steroids, second line immunosuppressive agents, and intra-vitreal/periocular steroids used to control intraocular inflammation.

Visual loss was defined as, the loss of two Snellen lines from baseline or a visual acuity of 6/12 or worse. Visual loss caused by macular scar, optic nerve changes, macular ischemia, and choroidal neovascular membrane was considered to be permanent visual loss. The amount of visual impairment was used to classify intensity of the disease which was defined as visual impairment during the episode of inflammation and attributed directly to the inflammatory process or its complications. It was graded into moderate or severe inflammation, with Snellen VA loss of 6/60 or worse taken as severe inflammation. Elevated intraocular pressure was defined as a persistently high intraocular pressure of more than 21 mm Hg as a direct result of inflammation. By definition it excluded steroid responders and any spikes of high intraocular pressure due to intra-vitreal or periocular corticosteroids.

### Controls

For comparison, the control population consisted of 282 healthy Caucasians aged 60 or older, who were admitted for age-related cataract surgery and had no previous ocular disease or immune-mediated disease.

### DNA extraction and genotyping

Genomic DNA was extracted from 10 ml EDTA-chelated peripheral whole blood, using commercial kits (Qiagen UK Ltd, Crawley, West Sussex, UK) according to the manufacturer's instructions. To identify potential SNPs, we compared multiple sequences for MCP-1, RANTES, CCR2, and CCR5 deposited in GenBank. When sequence differences were identified, these were regarded as potential SNPs and were further investigated. To verify the presence of each putative polymorphism, the SNPs were determined on a series of pooled Caucasian DNA samples using sequence-specific primers polymerase chain reaction (SSP-PCR) method [42]. SSP-PCR utilizes SSPs with 3'-end mismatches and identifies the presence of specific allelic variants through PCR amplification. In brief, each reaction contained 5 μl of the appropriate primer mix (allele-specific and control primers) and 8 μl PCR reaction mixture, containing 1X PCR buffer (Bioline, London, UK), 300 μM of each deoxynucleotide triphosphate (Bioline), 2 mM MgCl_2_, 0.32 U Taq polymerase (Bioline), and 0.01 to 0.1 μg DNA per well. The final volume of 13 μl was overlaid with 10 μl mineral oil in every PCR reaction. The DNA mixtures were heated at 96 °C for 1 min in the first round of denaturation and then subjected to 5 cycles of 25 s at 96 °C, 45 s at 70 °C, and 25 s at 72 °C; this was then followed by 21 cycles of 25 s at 96 °C, 50 s at 65 °C, 30 s at 72 °C; and finally 4 cycles of 30 s at 96 °C, 60 s at 55 °C, 90 s at 72 °C. All PCR amplifications were carried out under identical conditions in a PTC200 thermal cycle (model PTC200; Bio-Rad-MJ Research, Herts, UK). PCR products were then analyzed by electrophoresis on a 1% agarose gel. A positive reaction was defined as the presence of an allele-specific band of the expected size in conjunction with a control band. The absence of an allele-specific band in the presence of a control band was considered to be a negative reaction. The primers used are shown in Table 1.

**Table 1:** Primers used in detecting single nucleotide polymorphism in chemokine genes.

SNP	Chromosome	Identity	Primer (5'-3')	Product size (bp)
MCP-1 -2518A/G	17q11.2-q12	rs1024611	F: AAGTGGGAGGCAGACAGCTA/G	252
			R: CTGATAAAGCCACAATCCAGAG	
RANTES -403G/A	17q11.2-q12	rs2107538	F: CATGGATGAGGGAAAGGAGG/A	285
			R: GAGTCTCTGTCTCTCCCTCA	
RANTES -28C/G	17q11.2-q12	rs2280788	F: GCCCTTTATAGGGCCAGTTG/C	314
			R: GTCCTAACTGCCACTCCTTG	
CCR2 V64I	3p21	rs1799864	F: TTTTTGCAGTTTATTAAGATGAGGAC/T	808
			R: GAAGGCAGAAGGTGAATAGTTC	
CCR5 -59029G/A	3p21	rs2040388	F: ACTTCACATTAACCCTGTGC/T	712
			R: ACTGTCATTCAGCCCAATACC	
CCR5 32 bp deletion	3p21		F: GCTCTCATTTTCCATACAGTCAG/A	827/808
			R: TATACATAAGGAACTTTCGGAGTG	

The single nucleotide polymorphism (SNPs) were detected in 141 patients and 282 controls by sequence specific primers polymerase chain reaction (SSP-PCR) method. SSP-PCR utilizes SSPs with 3'-end mismatches and identifies the presence of specific allelic variants through PCR amplification.

### Statistical analysis

For each subject group, genotype frequencies were counted and the allelic and allele carriage frequencies calculated. All genotype frequencies in each population were tested for deviation from the Hardy-Weinberg equilibrium using the χ-square test. Data mining for significant associations was performed using Knowledge Seeker®, (Angoss softwares, Guilford, UK), and statistical calculations were performed with with statistical Package for social sciences SPSS V 12.0 (Chicago, IL). Confidence intervals were calculated at the 95% level and a value of p<0.05 was considered as significant. χ-square test was used to compare the genotypic and allelic frequencies in patients and controls. A correction for multiple comparisons was made using the Bonferroni method, adding the formula pc=p x n where pc represents the corrected value, p is the uncorrected value, and n is the numbers of comparisons performed. A multiple logistic regression model was used to determine association between various genotypes and the phenotypes.

For statistical purpose no attempt was made to divide the IPSU group into clinical subgroups as the groups would be meaningless for the statistical analysis. However whenever any association of a clinical sign, phenotype, or type of uveitis was noted with a SNP, multiple logistic regression model was used to correct the p value for the type of uveitis besides other parameters. Power calculations were performed using Quanto Version 1.0, which is specifically designed for use in genetic studies. Quanto is freely available from the following website (Hydra).

## Results

This study used 141 patients with IPSU. The mean age of onset of IPSU was 37.8 years. The female to male ratio was 1.4: 1. The demographic and clinical details are shown in Table 2. Among the groups the patients with pan uveitis had worse visual acuity compared to the others after first year of onset of disease (p=0.03) but the difference was not significant after the third year and onwards. There was also significant difference in the treatment given to the patients among the different groups. Patients with intermediate uveitis had more localized corticosteroid therapy (peri-ocular and intra-vitreal steroids) compared to other groups (p=0.01). Similarly permanent visual loss and high intraocular pressure were more common in pan uveitis patients compared to other patients. Hence when determining the association of these variables with genotypes, the statistical correction was done using multiple logistic regression. All genotype frequencies in the study conformed to Hardy-Weinberg equilibrium. The genotype frequencies of all six SNPs in PSU and controls are shown in Table 3.

**Table 2:** Demographic and clinical details of the 141 patients.

Follow up in years	Mean=6.8	Range=(1.5-42.6)
Sex	Males=59	Females=82
Laterality of disease	Bilateral=118	Unilateral=23
Age of onset (years)	Mean=37.82	Range=(5-70.5)
Recurrence (rate per year)	Mean=1.92	Range=(1-7)
MeanVisual impairment during inflammation	Mean=6/12	Range=(6/5-PL)
Best corrected vision after 18 months	Mean=6/9	Range=(6/5-HM)
Permanent visual loss	n=55	Mean=6/24, Range=6/12-HM
More than 10 mg of steroids for long term	n=51	
On second line of immuno-suppressants	n=51	
Cystoid macular edema	n=85	
Raised intra-ocular pressure	n=46	
Cataract	n=55	

141 patients with idiopathic posterior segment uveitis were included in this study. The follow up ranged from 1.5-42.6 years. This table summarizes the clinical details of the patients.

**Table 3:** Genotypic frequencies of chemokine single nucleotide polymorphisms in patients and controls.

Genotype		Patients n=(141)	Controls n=(282)
MCP-1 -2518 A/G	AA	58.2%	53.4%
	AG	36.9%	38.5%
	GG	5.0%	8.1%
RANTES -403 G/A	GG	61.0%	63.3%
	GA	34.8%	31.8%
	AA	4.3%	4.9%
RANTES -28 C/G	CC	94.3%	94.3%
	CG	5.7%	5.3%
	GG	0.0%	0.4%
CCR2 V64I	VV	82.3%	87.2%
	VI	16.3%	12.5%
	II	1.4%	0.4%
CCR5 -59029 G/A	GG	21.3%	22.3%
	GA	44.7%	48.6%
	AA	34.0%	29.1%
CCR2 32 bp deletion	wt/wt	77.3%	76.6%
	wt/del	20.6%	21.6%
	del/del	2.1%	1.8%

The genotypic frequencies of the six polymorphisms were compared between patients and controls. The frequencies are shown in percentage and as seen in the table there are no significant differences between the genotypic frequencies of patients and controls.

### Risk of developing uveitis

There was no significant difference in the genotypic frequencies between the controls and IPSU. Subgroup analysis also did not reveal any association of chemokine SNPs with any phenotype of IPSU apart from CCR2 64I allele, which was slightly higher in patients with intermediate uveitis (12%) when compared to the controls (7%; p=0.03, pc=0.09). The allelic frequencies in the subgroups are shown in Table 4.

**Table 4:** Allelic frequencies of chemokine single nucleotide polymorphisms in major subgroups of idiopathic posterior segment uveitis.

Genotype		Intermediate uveitis n=(77)	Pan uveitis n=(17)	Posterior uveitis n=(29)	Vasculitis n=(18)	Grand total n=(141)
MCP-1 -2518 A/G	AA	53%	71%	69%	50%	58%
	AG	43%	18%	28%	44%	37%
	GG	4%	12%	3%	6%	5%
RANTES -403 G/A	GG	58%	71%	59%	67%	61%
	GA	40%	24%	31%	28%	35%
	AA	1%	6%	10%	6%	4%
RANTES -28 C/G	CC	95%	88%	93%	100%	94%
	CG	5%	12%	7%	0%	6%
CCR2 V64I	VV	79%	76%	93%	83%	82%
	VI	18%	24%	7%	17%	16%
	II	3%	0%	0%	0%	1%
CCR5 -59029 G/A	GG	25%	6%	17%	28%	21%
	GA	40%	47%	66%	28%	45%
	AA	35%	47%	17%	44%	34%
CCR5 32 bp deletion	wt/wt	81%	76%	72%	72%	77%
	wt/del	17%	18%	28%	28%	21%
	del/del	3%	6%	0%	0%	2%

Idiopathic posterior segment uveitis was divided into four types on the basis of site of inflammation. Subgroup analysis did not reveal any association of chemokine SNPs with any phenotype of uveitis apart from CCR2 64I allele, which was slightly higher in patients with intermediate uveitis (12%) when compared to the controls (7%; p=0.03, pc=0.09). The genotypic frequencies are shown in percentages.

### Effect on phenotype

When the influence of genotypes on the clinical phenotype of the patients were compared, it was found that the MCP-1-2518 G allele was significantly associated with a younger age of onset of disease. The mean age of diesase onset in patients with the AA genotype was 41 years compared to 33 years in patients with the G allele carriage (pc=0.003). This difference was still significant after correcting for type of IPSU pc=0.006. It was also noted that the RANTES-403 polymorphism was associated with the disease severity (VA impairment of 6/60 or less during an episode of active inflammation), and this difference was still significant when corrected for uveitis type, disease duration, and treatment received. Severe inflammatory episodes were seen in 38% (33/86) of the patients with the GG genotype as compared to 20% (11/55) in patients with the A allele carriage. (pc=0.04 OR=0.40, 95% CI=0.18-0.88). The G allele was also significantly associated with worst visual acuity in the affected eye. As shown in Table 5 patients with the GG genotype had a worse mean visual acuity as compared to carriers of the A allele after a ten year period.

**Table 5:** Comparison of mean visual acuities between RANTES -403 GG and RANTES -403 GA/AA over a ten-year period.

Genotype	Mean VA at year 1	Mean VA at year 2	Mean VA at year 3	Mean VA at year 5	Mean VA at year 10
RANTES GG	0.5103	0.4824	0.4817	0.4155	0.3844
RANTES GA/AA	0.7140	0.6783	0.6726	0.6213	0.5422
P value (2-tailed)	0.002	0.013	0.016	0.026	0.056

The G allele of RANTES -403 SNP was significantly associated with worst visual acuity in the affected eye. As shown in this table patients with the GG genotype had a worse mean visual acuity as compared to carriers of the A allele. The difference was greater in early years but even after 10 years of onset of disease the patients with A allele carriage had better mean visual acuity.

The CCR5 del. 32 was found to be associated with visual outcome even after correction for disease phenotype and treatment. No patient with a final VA of 6/12 or worse was found to be homozygous for del. 32. The mean VA after 18 months (which was the minimum follow-up period) in patients with wild-type genotype was 6/9 where as it was 6/6 in patients with del. 32 allele (pc=0.04). The frequency of the 32 del. allele in patients with VA 6/9 or better was 15% compared to 3% in the group with a VA of 6/12 or worse (p=0.01, pc=0.028) corrected for age, type of uveitis, treatment and duration of disease. CCR2 V64I, which is strongly linked to the CCR2 32 bp deletion, also showed association with visual outcome. The patients with CCR2 64VV had a mean visual acuity of 6/9 after 18 months compared to 6/15 among patients with 64I allele carriage. After correction for uveitis type, treatment, and patient age, the difference was still significant (pc=0.04). It was also noted that among carriers of the I allele 60% (15/25) of the patients developed elevated intraocular pressure compared to 26.7% (31/116) of the patients with the wild-type genotype (p=0.001, OR 4.11; 95%CI: 1.67-10.11). After correction for the treatment, patient age, disease type, and duration, the pc was 0.007. CCR5 -59029 SNP was associated with the need for persistent corticosteroid therapy of more than 10 mg per day to control inflammation. We found 20% (6/30) of the patients with the wild type GG allele had received long-term corticosteroid treatment compared to 39.6% (44/111) of patients with A allele carriage p=0.046 which remained significant after correcting for duration of disease and type of uveitis p=0.05. The summary of these finding is shown in Table 6.

**Table 6:** Summary of significant associations between the chemokine polymorphisms and idiopathic posterior segment uveitis.

Phenotype	Genotype		p value
	MCP-1 -2518 AA n=82	MCP-1 -2518 AG & GG n=59	
Mean age of onset of *IPSU	41 years	33 years	pc=0.003
	RANTES -403GG n=86	RANTES -403 GA & AA n=55	
Severe inflammatory episodes	38% (33)	20% (11)	pc=0.04
	CCR5 wt/wt n=109	CCR5 del/wt & del/del n=32	
Meanv Visual acuity after 18 months	6/9	6/6	pc=0.028
	CCR2 VV n=116	CCR2 VI & II n=25	
Meanv Visual acuity after 18 months	6/9	6/15	pc=0.04
Raised intra-ocular pressure	27% (31)	60% (15)	pc=0.007
	CCR5 -59029 GG n=30	CCR5 -59029 GA & AA n=111	
On long term corticosteroid treatment	20% (6)	40% (44)	pc=0.051

This table shows the effect of chemokine SNPs on the phenotype of idiopathic posterior segment uveitis. Logistic regression model was used to perform the statistical calculations. Results show that chemokine SNPs can affect the phenotype of idiopathic posterior segment uveitis and can be predictors of severity and complications. Asterisk indicates idiopathic posterior segment uveitis.

## Discussion

In this study, we investigated the potential significance of the CC chemokines MCP-1 and RANTES and their receptors CCR2 and CCR5 in immune-mediated PSU by analyzing the association of genetic polymorphisms of these four genes with the clinical outcome in patients with PSU. The aim of the study was to see whether these functional SNPs could affect the clinical outcome of PSU independent of uveitis type. The results show that polymorphisms in chemokine genes can affect the disease phenotype in PSU.

The MCP-1-2518 G mutation is known to increase the expression of the MCP-1 gene [30]. Many studies have shown an association of this allele with immune-mediated diseases [43,44] and anterior uveitis [45]. The frequency of G allele in the controls was 23% which is similar to previous reported studies [31,46]. No significant difference was noted in the genotypic frequencies between the control and IPSU populations. Interestingly however, we found that the carriage of the G allele was associated with disease development at an earlier age compared to patients with no G allele. A multiple logistic regression analysis showed that the G allele was not correlated to any specific disease group, and hence after correction for type of uveitis the association was still significant. Although it is difficult to say how this allele can affect the age of onset, it is important to note similar results have been documented in other diseases that also show association of MCP-1-2518 G allele with early onset of disease [47].

Enhancement of RANTES gene expression has been observed with the presence of the -28G allele and the -403A allele [32,33]. The -403A and -28G alleles have been associated with various immune-mediated diseases [48-52] but we did not detect any significant difference in the genotypes between the IPSU patients and controls. The frequencies of both SNPs in patients and controls were similar to other published studies [32,51]. As shown by other studies [53], the two SNPs were in linkage disequilibrium. RANTES -28 G/G was not found in subjects with -403 G allele, and RANTES -403G/G was not observed in subjects with -28 G allele. PSU patients with the -403A allele developed less intense inflammation (as judged by amount of visual impairment during an inflammatory episode) compared to patients with the wild-type genotype. We also observed that patients with A allele carriage had better visual acuity compared to GG genotype at least for 10 years after the onset of uveitis. It appears that the -403A allele, which is associated with increased RANTES expression, has a protective effect in patients with PSU. This is in contrast with other studies where the A allele was found to be associated with increased disease susceptibility and severity [47-50]. Recently Sonoda et al. [54] showed that in experimental autoimmune uveitis (EAU), which is the animal model for posterior uveitis, RANTES produced by ocular macrophages appears to have a suppressive effect. In another study [55] RANTES levels remained high during the recovery phase of autoimmune anterior uveitis associated with experimental autoimmune encephalomyelitis (EAE), and treatment with anti-RANTES antibodies was not effective in suppressing the uveitis. These studies show that decreased levels of RANTES may be associated with prolonged severe inflammation, and hence the -403A allele, which increases the RANTES expression, appears to help in its control.

With CCR2 V64I SNP we noticed a nonsignificant trend of increased frequency of 64I allele in patients with intermediate uveitis as compared to controls (p=0.03, pc=0.09). This 64I allele was also noted to be the predictor of elevated intraocular pressure. Patients with this allele had worse VA after 18 months when compared to patients with the wild-type genotype.

In the CCR2 V64I polymorphism, although this amino acid change is conservative, various studies have shown that the 64I allele may be a protective factor in immune-mediated diseases like sarcoidosis and multiple sclerosis [36,56,57]. However the authors were unable to explain the process that would confer protection against disease with the 64I mutation, as no functional changes have yet been identified [58].

Our data suggested that the 64I mutation could be an independent risk factor for the development and complications of uveitis in Caucasians. This is in contrast with other studies of systemic immune-mediated diseases [36,56,57], where whenever the association is present, this 64I allele has found to be protective. However, at least in age related macular degeneration this 64I allele has been implicated as a risk factor [59]. One explanation for this is that, the 64I mutation has been noted to increase the stability of the CCR2A isoform [60]. This in turn leads to higher cell surface expression of CCR2A (but not CCR2B isoform) as compared to the V64 allele. These two isoforms are alternatively spliced variants of a single CCR2 gene. Although CCR2B is normally present in greater amount, it is possible that increased CCR2A isoform levels due to 64I mutation cause the CCR2A isoform to play a greater role. Given the lack of enough evidence of functional effects of V64I mutation it is more likely that the effects are seen as a result of a yet to be identified gene linked to CCR2-64I that encodes a polymorphic site that plays a role in the immune response in IPSU.

As noted by other studies [61], the CCR2 V64I was tightly linked with CCR5 -59029, and del. 32. 64I homozygosity was not found in subjects with the CCR5 -59029G allele, while -59029G homozygosity was not observed in subjects with the CCR2 64I allele. Also, subjects homozygous for CCR2 64I were never homozygous for CCR5 del. 32. The del. 32 in CCR5 is common in Caucasians as compared to other ethnic groups and plays an important role against human immunodeficiency virus (HIV) infection and progression to acquired immune deficiency syndromes (AIDS). However, its role in immune-mediated disease is not clear. The presence of the deletion results in a truncated form of the functional receptor, and homozygotic carriers of this mutation fail to express CCR5 on their cell surface. As in other studies, there was a strong linkage between CCR5 del. 32 and CCR5 -59029 in our study. All patients with del/del phenotype were homozygous for the -59029 G allele. Similarly all AA homozygous for -59029 were also homozygous for the wild-type allele. In our study there was no association found between these two SNPs and the risk of developing PSU. However the del. 32 was associated with a better final visual acuity after 18 months. The mean final VA of patients having the del. 32 allele was better than patients with no mutation. If the del. 32 leads to under expression of the CCR5 receptors, the presence of this mutation might confer protection against a more severe form of inflammation, and thus patients with this mutation would be expected to have a better visual outcome.

CCR5-59029 A allele is a pro-inflammatory mutation. It has been shown to be an independent risk factor for diabetic nephropathy in patients with type 2 diabetes [61], and acute kidney graft rejection in patients with a donor kidney [62]. We found that the A allele was associated with the continuous need for systemic corticosteroids to control the inflammation, and we also noted a non-significant trend of higher A allele frequency (53%) among patients with intermediate uveitis requiring systemic steroids (n=47) compared to 33% in patient not requiring systemic steroids (n=30; p=0.02, pc=0.06 suggesting that this polymorphism has an effect on the severity of the inflammatory process). These results show polymorphisms that decrease CCR5 expression improve the visual outcome. On the other hand, polymorphisms that decrease RANTES, which is a ligand of CCR5, are associated with poor visual outcome. These two results seems contradictory, but it should be noted that CCR5 is not just a receptor of RANTES, but also of MIP-1α and MIP-1β [63]. CCR5 shows maximum activity toward MIP-1α, and this ligand plays an important role in uveitis [64]. So while CCR5 may be involved in recruiting the activated T cells and macrophages toward the site of uveitis through MIP-1α, RANTES may be involved in the immunomodulation and suppression of uveitis. CCR2 and CCR5 genes are located on chromosome 3p21, and the SNPs studied here also show a strong degree of linkage. These moderate associations noted between IPSU and CCR2 and CCR5 SNPs indicate that a locus for genetic susceptibility for IPSU may be located on chromosome 3 in close proximity to these SNPs.

In conclusion, this study suggests the involvement of certain chemokine gene polymorphisms that can influence disease outcome in patients with IPSU. We propose that RANTES -403G/A SNP, and polymorphisms in CCR5 and CCR2 genes can affect the outcome of the disease. Further studies will be necessary to determine whether patients with alleles indicative of more severe disease may benefit from earlier treatment with more aggressive therapy to try and prevent visual loss.
